# Rift Valley Fever in Small Ruminants, Senegal, 2003

**DOI:** 10.3201/eid1111.050193

**Published:** 2005-11

**Authors:** Véronique Chevalier, Renaud Lancelot, Yaya Thiongane, Baba Sall, Amadou Diaité, Bernard Mondet

**Affiliations:** * Centre International de Recherche Agronomique pour le Développement, Montpellier, France; † Ambassade de France, Antananarivo, Madagascar; ‡ Institut Sénégalais de Recherche Agricole, Dakar-Hann, Senegal; § Direction de l'Élevage, Dakar, Senegal; ¶ Institut de Recherche pour le Développement, Dakar-Hann, Senegal

**Keywords:** Rift Valley fever, disease surveillance, serologic incidence, temporary ponds, small ruminants, Senegal, research

## Abstract

During the 2003 rainy season, the clinical and serologic incidence of Rift Valley fever was assessed in small ruminant herds living around temporary ponds located in the semi-arid region of the Ferlo, Senegal. No outbreak was detected by the surveillance system. Serologic incidence was estimated at 2.9% (95% confidence interval 1.0–8.7) and occurred in 5 of 7 ponds with large variations in the observed incidence rate (0%–20.3%). The location of ponds in the Ferlo Valley and small ponds were correlated with higher serologic incidence (p = 0.0005 and p = 0.005, respectively). Rift Valley fever surveillance should be improved to allow early detection of virus activity. Ruminant vaccination programs should be prepared to confront the foreseeable higher risks for future epidemics of this disease.

## Abstract 2

Serologic incidence was estimated at 2.9%.

Rift Valley fever (RVF) is an arbovirosis caused by a phlebovirus (*Bunyaviridae*). In ruminants, RVF causes mass abortions and deaths in newborn kids and lambs. Human disease is often limited to a flulike syndrome, but severe forms have been reported (*1*). In West Africa, domestic ruminants are the main hosts of the virus, which is transmitted between animals by mosquitoes, particularly those belonging to the *Culex* and *Aedes* genera (*2**,**3*). Transmission is mostly horizontal, but a vertical mode was described for some *Aedes* species. Human cases are mainly caused by virus exposure after abortion or slaughtering of viremic animals (*1*).

A large RVF epidemic occurred in 1987 in southern Mauritania, with >200 reported human deaths (*4*). In the following years, several animal and human outbreaks occurred in Mauritania, Senegal, which emphasizes the need for understanding and modeling the risk for RVF in this region before implementing more efficient surveillance and control measures (*5**–**7*). For this purpose, a survey was conducted in the pastoral area of the Ferlo in northern Senegal.

During the rainy season, this agro-ecosystem depends on the availability of surface water in temporary ponds that are flooded after the first rainfalls. These ponds also constitute a favorable habitat for RVF vectors. Previous studies showed that Barkedji, a village located in the central part of the Ferlo, was an area with active viral circulation (*5**,**6*). The purpose of this study was to assess RVF activity in the area of Barkedji during the 2003 rainy season and to identify risk factors for its transmission to livestock.

## Materials and Methods

### Study Area

**Figure 1:**
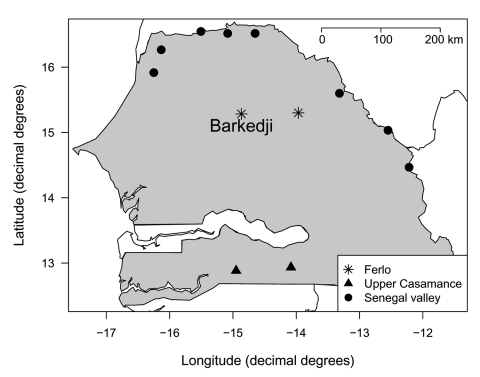
Location of the study of Rift Valley fever serologic incidence (Barkedji) and sentinel herds of the national surveillance system during the 2003 rainy season in Senegal.

**Figure 2:**
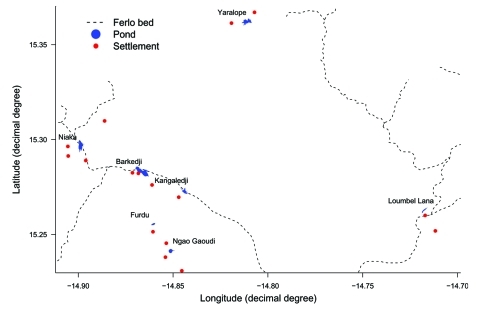
Location of ponds and settlements for the study of Rift Valley fever serologic incidence in 610 small ruminants during the 2003 rainy season in the Barkedji area, Senegal.

The survey area (Figures 1 and 2) was a 40-km diameter circle centered on the village of Barkedji (14°52´W, 15°16´N). The shrubby vegetation and hot, dry climate were typically Sahelian, with annual rainfall ranging from 300 to 500 mm, which occurred from July to October. The soil was made of a lateritic crust partially covered by flattened sandy dunes, stabilized by the vegetation. This plateau was eroded by a former effluent of the Senegal River, the Ferlo, which stopped flowing at the end of the last humid Saharan period (Neolithic era). The erosion left a large, fossil valley that crosses the study area from east to west with former effluents coming from the north and the south.

A low-input, extensive livestock-production system was adopted by both settled and transhumant farmers in the Ferlo. They used natural grasslands and surface water as much as possible. During the rainy season, temporary ponds—many in the Ferlo Valley—were flooded. These water resources enabled ruminants to use the surrounding grasslands. Transhumant farmers left the crop-farming regions of Senegal, where they spent the dry season, and converged on the Ferlo to benefit from these resources. The farmers gathered in compounds on the basis of family and ethnic relationships. They left the Ferlo at the end of the rainy season, as the temporary ponds progressively dried up.

### Disease Surveillance by the National Veterinary Services

The National Veterinary Services had been surveying the occurrence of RVF in Senegal since the 1987 epidemic. Coordinated by the National Veterinary Services' epidemiologic unit, the surveillance system involved the National Veterinary Research Laboratory (ISRA-LNERV), the Pasteur Institute of Dakar, and field veterinary services (*8*).

Disease surveillance was activated during the rainy season. Farmers were asked to report a high incidence of abortions in ruminants (cattle, sheep, and goats) to veterinary officers and private veterinarians. When such alerts arose, veterinary officers had to visit the suspected herds, carry out an epidemiologic survey, sample blood of females who had aborted, and collect fetuses. Biological samples were sent to ISRA-LNERV, where serologic tests were conducted. When relevant, reverse transcription–polymerase chain reaction (RT-PCR) and virus isolation were conducted at Pasteur Institute of Dakar.

Twelve veterinary posts were selected to perform serologic surveillance along the Senegal River Valley, in the pastoral area of Ferlo, including Barkedji, and in Upper Casamance (the southern, forested area), because of the previous RVF outbreaks in these places (Figure 1). The goal of this survey was to detect the background incidence of RVF. Sentinel herds (sheep and goats) were identified within the influence area of the selected posts. In the sentinel herds, 30 animals were ear-tagged, and their blood was sampled before the beginning of each rainy season and 2 or 3 times during the rainy season, depending on the length of the rainy season and observed activity of mosquitoes. A serum neutralization test was performed to detect anti-RVF neutralizing antibodies by using Vero monolayer cells infected with a viral suspension of 106.5 PFU/mL of the attenuated RVF virus Smithburn strain. A positive result was defined as a serum sample that showed a lack of cytopathogenic effect at a dilution of 1:160 (*9*).

### Assessment of Transmission Risk

The serologic incidence of RVF was estimated around selected temporary ponds of the Barkedji area. Incidence was measured by the frequency of seroconversions (change from negative to positive status) in small ruminants from the beginning to the end of the rainy season. Interviews with the farmers showed that their criteria for choosing the pond were related to its size. Large ponds were preferred because they remained flooded longer than smaller ponds. Moreover, farmers tended to settle close to the ponds because they also used the water for family needs.

Seven ponds were selected according to their location (inside or outside the Ferlo riverbed) and their size. Size was computed from their perimeters, recorded with a 12-channel global-positioning-system (GPS), after a series of heavy rainfalls, i.e., when the watered surface of each pond was at its maximum.

At the beginning of the rainy season, meetings were organized with the farmers settled around each of the 7 ponds to explain the goal of the study. The decision to participate in the survey was made at the compound level, which comprised several families and herds. Sixteen compounds were selected, and their geographic position was recorded with a GPS to compute their distance from the pond, defined as the minimum distance between the compound and the perimeter of the related pond. This risk factor was chosen because ruminants spent the night in pens located in the compounds. Because *Aedes* and *Culex* vectors of RVF virus have a crepuscular or night activity, RVF transmission probably occurs within these pens. The location of ponds and compounds is shown in Figure 2.

The minimum number of animals to be tagged and sampled was set at 30 in each compound, to detect at least 1 seroconversion, with a 95% confidence level, in the case of a 10% serologic incidence. Sampling was performed in August for the first occasion and from mid-November to mid-December for the second (Table 1). Blood samples were centrifuged and serum specimens were stored at –20°C until they were tested at ISRA-LNERV for anti-RVF antibodies with the serum neutralization test described above. Farmers who participated in the survey were asked to report abortions that occurred in ruminants, whatever their involvement in the serologic study.

**Table 1:** Timeline (month/day) of the study of serologic incidence of Rift Valley fever in small ruminants, Barkedji area, Senegal, 2003 rainy season*

Pond name	First sampling date	Second sampling date
Niaka	08/29–08/30	12/12–12/18
Barkedji	08/02–08/08	12/04–12/09
Loumbel Lana	08/06–08/09	12/06–12/07
Furdu	08/28	11/11–11/12
Ngao Gaoudi	08/10–08/28	12/12–12/15
Kangaledji	07/31–08/01	12/06–12/12
Yaralope	07/29–08/01	12/14–12/17

*At the pond level (*n*_1_ = 7) and at the compound level (*n*_2_ = 16); N = 610 small ruminants.

### Data Analysis

Serologic incidence data were analyzed by using logistic-regression mixed models (LRMM) (*10*). Incidence was the response, aggregated at the compound level (i.e., 1 line per compound in the dataset). The pond was included as a random effect in the models. This strategy allowed estimates of both population-level mean (overall incidence) and pond-specific means.

Three main effects and their interactions were considered in the fixed part of the model: 1) the location of the pond (inside or outside the Ferlo riverbed), 2) its size expressed in hectares (ha) and centered on the surface of the smallest pond, and 3) the distance between the pond and the compound, expressed in hectometers (hm) and centered on the smallest observed distance. The explanatory variables are displayed in Table 2. For surface and distance, only linear effects were considered.

**Table 2:** Variables selected to explain serologic incidence of Rift Valley fever in small ruminants, Barkedji area, Senegal, 2003 rainy season*

Pond name	Pond location	Surface (ha)	Compound code	Distance: pond-compound (hm)
Niaka	Ferlo bed	10.1	BEL	17.8
			NIA	6.7
			NIK	6.1
Barkedji	Ferlo bed	17.9	BK1	0.6
			BK2	2.8
			BK3	5.1
Loumbel Lana	Ferlo bed	1.8	DIA	12.6
			LOU	2.1
Furdu	Outside Ferlo bed	1.6	FUR	3.9
Ngao Gaoudi	Outside Ferlo bed	4.2	GAW	4.0
			NG2	3.4
			NGA	11.9
Kangaledji	Ferlo bed	4.7	KAN	3.9
Yaralope	Outside Ferlo bed	9.4	YA1	5.8
			YA2	4.8

*At the pond level (n_1_ = 7) and at the compound level (n_2_ = 16); N = 610 small ruminants; hm, hectometers.

No prior information was available to determine the most plausible model. Therefore, all the possible models with these 3 main effects and their 2- and 3-way interactions were fitted. To avoid the problem of multiple model comparison (e.g., with the likelihood ratio test), the Bayesian information criterion (BIC) was used to select the most plausible model (11,12): BIC = –2 log(ML) + *k* log(*n*), where ML was the maximized likelihood, *k* was the number of parameters in the model, and *n* was the number of observations (number of compounds). For this information criterion, the best model was the one with the lowest value. A database management system designed for herd follow-up was used to enter and store the data (*13*). R software was used for data analysis and graphs (*14*).

## Results

### Disease Surveillance

During the 2003 rainy season, no outbreak of RVF was confirmed in the Barkedji area by the national surveillance system. However, 76 abortions were reported in small ruminants by farmers in this area, either to the Barkedji veterinary officer or to research staff. Eleven abortions occurred among animals included in the serologic survey. The sera of 2 of these ewes, which lived near the Loumbel Lana pond (Figure 2), were positive for RVF with the serum neutralization test. In Furdu (Figure 2), farmers reported 7 abortions in ewes that were not involved in the serologic survey. Blood samples were taken from these ewes, and 2 serum samples were positive for RVF. In both cases (the national surveillance system and the farmers involved in the serologic survey), abortions were reported late. Consequently, no sample was obtained from the fetuses or from the fetal envelopes for RT-PCR test or virus isolation.

### Assessment of Transmission Risk

A total of 610 sheep and goats were sampled on the first occasion. Three ewes' serum specimens were positive with the serum neutralization test (Furdu, Yaralope, and Niaka). They were discarded from the incidence analysis. On the second occasion, 379 animals were sampled (38% of the initial samples were lost to follow-up) (Table 3). At the pond level, the maximum rate of missing data was observed in Niaka (62.7%), and the minimum rate was found in Furdu (2.5%). At the compound level, the lost-to-follow-up rate ranged from 2.5% (Furdu) to 100.0% (Niaka). In this case, the whole compound left the area before the second sampling occasion. For all subsequent analyses, the denominator of incidence probabilities was computed as the initial number of sampled animals minus half the number that were lost to follow-up. This correction assumed that lost-to-follow-up processes were independent from RVF incidence. This assumption was assessed both graphically (graph not shown here) and by computing a logistic regression of the incidence rate against the proportion of ruminants lost to follow-up. A weak positive trend was found, but the slope coefficient was not significantly greater than zero (p = 0.16).

**Table 3:** Sample size, lost to follow-up, and observed serologic incidence of Rift Valley fever in small ruminants, Barkedji area, Senegal, 2003 rainy season*

Pond	Compound code	Initial size	Lost to follow-up	Serologic incidence
Barkedji	BK1	20	0	0
	BK2	50	28	0
	BK3	30	15	0
Furdu	FUR	40	1	1
Kangaledji	KAN	86	34	14
Loumbel Lana	DIA	12	7	2
	LOU	89	69	1
Ngao Gaoudi	GAW	40	10	1
	NG2	30	3	1
	NGA	20	2	1
Niaka	BEL	17	1	0
	NI4	20	20	-
	NIA	37	10	4
	NIK	20	18	1
Yaralope	YA1	20	11	0
	YA2	79	2	0

*For 7 ponds and 16 compounds; N = 610 small ruminants.

The observed serologic incidence rate of RVF was 5.4%, with large within- and between-pond differences (Tables 3 and 4), ranging from 0.0% in Barkedji and Yaralope ponds to 20.3% in Kangaledji pond (Table 4). The average incidence rate, estimated from the intercept-only LRMM, was 2.9% (95% confidence interval 1.0–8.7). Observations and model predictions both indicated that RVF transmission occurred in 5 of 7 ponds in the study area during the 2003 rainy season and that the transmission probability differed widely from pond to pond.

**Table 4:** Observed and fitted serologic incidence rate of Rift Valley fever at the pond level in small ruminants, Barkedji, Senegal, 2003 rainy season

Pond	Observed	Fitted*
Barkedji	0.0	0.9
Furdu	2.5	2.7
Kangaledji	20.3	18.6
Loumbel Lana	4.8	4.4
Ngao Gaoudi	3.6	3.5
Niaka	8.4	7.5
Yaralope	0.0	0.8

*Serologic incidence was fitted with an intercept-only mixed-effect logistic regression model, with the pond as the random effect. N = 610 small ruminants.

**Figure 3:**
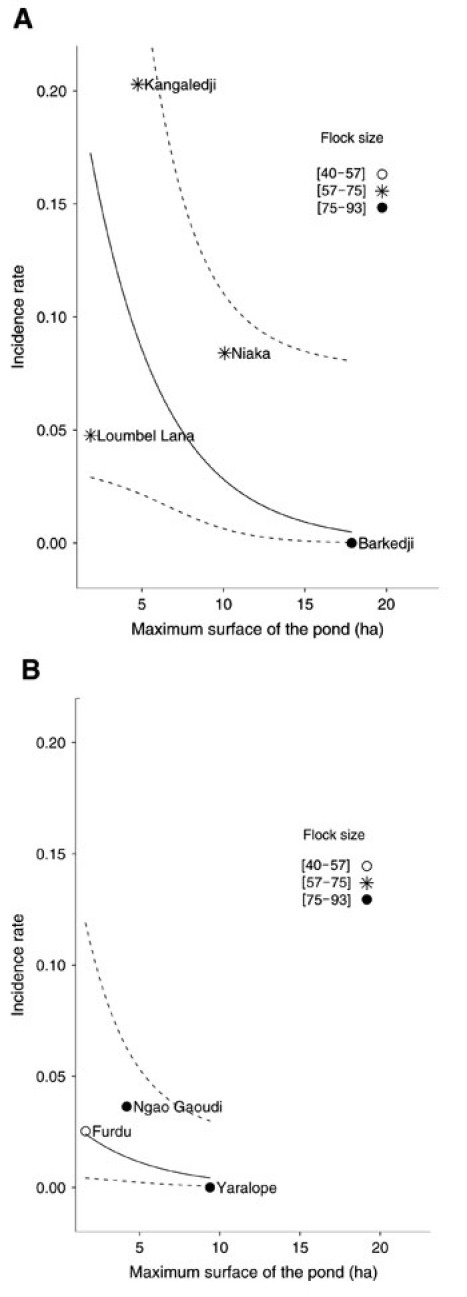
Serologic incidence rate of Rift Valley fever in small ruminants (N = 610), according to the location of the pond (A, in Ferlo River bed; B, outside Ferlo River bed) and its maximum surface during the 2003 rainy season in the Barkedji area, Senegal. Points indicate observed pond-level serologic incidence. Solid line indicates population mean of the serologic incidence estimated with the best Bayesian information criterion mixed-effect logistic regression model. Dashed lines indicate pointwise 95% confidence interval corresponding to these estimates. ha , hectares.

Comparison of the 19 possible models is shown in Table 5. The best model according to BIC was the incidence as a function of surface and pond location for the fixed effects. The coefficients of the intercept-only and the best BIC model are shown in Table 6. Fixed-effect coefficients of this model were significantly different from zero (surface p = 0.04; Ferlo p = 0.03). The 3-fold reduction of the variance of the random effect between the intercept-only (variance = 1.75) and the best BIC model (variance = 0.57) indicated that the within-pond correlation of the results was well accounted for by the fixed effects. The population mean of the RVF serologic incidence, as predicted by the location and the surface of the ponds, is displayed in Figure 3. This figure shows that the serologic incidence was higher inside the Ferlo riverbed than outside, and that smaller ponds encountered a higher RVF incidence than larger ponds.

**Table 5:** Bayesian information criteria for 19 mixed-effect logistic regression models of Rift Valley fever serologic incidence at 15 compounds, in small ruminants,* Barkedji area, Senegal, 2003 rainy season

Fixed model	BIC†
Surface‡ + Ferlo§	32.3
Surface + distance¶ + Ferlo + surface × distance	33.0
Intercept-only model	33.2
Surface + distance + surface × distance	33.6
Surface	33.6
Ferlo	34.2
Surface + Ferlo + distance	34.5
Surface + Ferlo + surface × Ferlo	34.6
Surface + Ferlo + distance + surface × Ferlo + surface × distance	35.3
Distance	35.7
Surface + distance + Ferlo + surface × distance +distance × Ferlo	35.7
Surface + distance	36.0
Distance + Ferlo	36.7
Surface + Ferlo + distance + surface × Ferlo	36.7
Ferlo + distance + surface + distance × Ferlo	37.1
Surface + Ferlo + distance + surface × Ferlo + surface × distance +distance × Ferlo	38.0
Distance + Ferlo + distance × Ferlo	39.3
Surface + Ferlo + distance + surface × Ferlo + distance × Ferlo	39.3
Surface + Ferlo + distance + surface × Ferlo + surface × distance + distance × Ferlo + surface × Ferlo × distance	39.8

*With the pond as the random effect. N = 610 small ruminants. †Bayesian information criterion. ‡Surface (hectare) centered on the smallest observed surface (1.6 hectare). §Location of the pond: in the Ferlo bed (reference category) or outside the Ferlo bed. ¶Distance from the compound to the pond (hectometers) centered on the shortest observed distance (0.6 hectometers).

**Table 6:** Parameters of the intercept-only and the best BIC mixed-effect binomial model of Rift Valley fever serologic incidence in small ruminants, Barkedji area, Senegal, 2003 rainy season*

Term	Parameter	Standard error	Z*	p†
Intercept-only mixed-effect model				
Intercept	–3.49	0.58	–5.99	2.1 × 10^–9^
Variance of the random effect‡	1.75	–	–	–
Best mixed-effect model according to information criteria				
Intercept	–1.62	0.67	–2.41	0.02
Surface§	–0.19	0.09	–2.08	0.04
Ferlo¶	–1.95	0.88	–2.21	0.03
Variance of the random effect‡	0.57	–	–	–

*Ratio of the parameter over its standard error. N = 610 small ruminants; BIC, Bayesian information criterion. †P(*x* > |Z|) assuming a standard-normal distribution for *Z;* straight vertical bars indicate absolute value of *Z*. ‡Pond-related random effect associated with the intercept. §Surface (hectare) centered on the smallest observed surface (1.6 hectare). ¶Location of the pond: in the Ferlo bed (reference category) or outside Ferlo bed.

## Discussion and Conclusion

### Disease Surveillance

In Senegal, 5 outbreaks were reported by the national RVF surveillance network in 2003 (*15*). They occurred in the Senegal River Valley; none was reported in the Ferlo. However, our serologic results showed that RVF virus actively circulated in the Barkedji area in 2003 and that clinical cases probably occurred in small ruminants.

RVF was detected at the national level. However, the surveillance system was not sensitive enough to detect all outbreaks of RVF. The disease warning was issued in November, i.e., at the end of the rainy season, when ponds dried up. At this time, most transhumant farmers had already left the Ferlo (and other pastoral areas) to join their dry-season settlement. Therefore, a high risk for virus dissemination existed before the warning was given.

A more efficient system should provide evidence of virus circulation at the beginning of the rainy season (July or August). Because Senegal has a long experience in RVF surveillance and outbreak investigations, defining a few hot points, e.g., along the Senegal River valley and in the Ferlo, should be possible; more stringent surveillance could be implemented in these locations, with RT-PCR, virus isolation on entomologic and ruminant samples, or both.

In addition, preventive measures should be considered, such as the vaccination of ruminants to break the amplification cycle of the virus. In July, the beginning of the rainy season, cattle, sheep, and goats are not pregnant. Births occur before the rainy season (May–June for cattle, earlier for small ruminants), and their reproduction cycle is stopped during the hot, dry season because of lack of energy and protein in their diet. Therefore, the residual pathogenicity of the Smithburn vaccinal strain (i.e., a risk for abortion in pregnant ewes) should not be a problem. Moreover, July is the period when farmers usually vaccinate ruminants against anthrax, black leg, botulism, and pasteurellosis. The addition of RVF to this list of recommended vaccines should thus serve the interests of farmers.

These prevention measures will become more important to consider during coming years. Like each Muslim feast, Aïd El Kebir is determined according to the lunar calendar. Therefore, for a given year, this date occurs 10 or 11 days earlier than in the former solar year. At the occasion of this feast, tens of thousands of sheep are slaughtered on the same day, which implies massive animal movements and potential spread of the disease. In addition, slaughtering happens at home, with a high risk of spreading of the virus to humans if the sheep are viremic. In 2005, Aïd El Kebir occurred on January 19. In coming years, the feast will occur during the high-risk period of RVF occurrence.

### Assessment of Transmission Risk

The use of the serum neutralization test limited the risk for cross-reaction with other phleboviruses, but the sensitivity of the analyses was low. This feature of the serum neutralization test probably resulted in underestimates of the incidence rate. Moreover, the incidence results were difficult to compare with those of other prevalence surveys undertaken in Mauritania or Senegal (*7**,**16*).

The overall serologic incidence was rather low (5.4%), but RVF transmission occurred in a large proportion of the temporary ponds (5/7) in the study area during the 2003 rainy season. Earlier works suggested that Barkedji was an area of endemic activity for RVF virus (*6*). The serologic results observed in this study were compatible with this hypothesis. Vertical transmission of the virus in *Aedes* mosquitoes might explain the maintenance of RVF infection in this region. The alternative, and nonexclusive, hypothesis is that RVF virus is introduced in Barkedji by ruminants that are seasonally moved. Confirmatory studies should involve a follow-up survey of transhumant cattle in their dry- and rainy-season settlements, to assess where transmission primarily occurs.

Serologic incidence differed from pond to pond: Barkedji and Kangaledji ponds (Figure 2, Table 4) had different RVF transmission rates, although they were close to each other. This result corroborates previous findings from the same area, which showed that the exposure to *Aedes vexans* bites, and consequently the risk for RVF transmission, was spatially heterogeneous (*17*). This previous study also suggested that because very few *Aedes* mosquitoes were captured near the Barkedji pond, it had a low risk for RVF transmission. We confirmed this finding.

The lack of protective effect of distance between the pond and compound was probably related to the low range of investigated distances. This range reflects the actual situation, i.e., that farmers like to settle close to ponds. This finding offers few practical recommendations. Even when farmers increased the distance (within the observed distance range) between settlements and ponds in the Ferlo, their herds were not protected against mosquito bites and RVF.

The Ferlo Valley was densely populated by RVF hosts during the rainy season. Moreover, the rather dense tree and grass cover offered a large choice of resting sites for mosquitoes. These favorable conditions for the amplification of RVF virus probably explain why the incidence rate was higher in the Ferlo bed than outside it. Although most ponds of interest for the livestock were located in the Ferlo bed, some outer ponds, like Yaralope and Furdu (Figure 2), were used by farmers because of the large available space and, according to them, the lower risk for sheep schistosomiasis. The optimal use of these outer ponds should thus be encouraged.

The lower incidence observed around large ponds might be related to the predominance of *A. vexans* in the transmission of RVF during the 2003 rainy season. The eggs of this species are laid on the wet soil of the pond banks, and their desiccation is needed before they hatch, when they are watered again. They can survive for several years in the dried mud (*18*). When the ponds are flooded again, a mass hatching of mosquito eggs occurs, and adult neonates appear 4–8 days later (*19*). In the study area, larger ponds were also deeper than smaller ones. Once watered, these ponds exhibited slower and more limited changes in the flooded surfaces than did the smaller and shallow ponds, which resulted in fewer mosquitoes hatching and a lower transmission risk. However, the relationship between pond size and incidence might be reversed in the case of RVF transmission by *Culex* mosquitoes, which need water all during their development cycle (*20*). Previous studies have shown that *Culex* species were sometimes predominant in the Barkedji area, depending on the rainfall patterns during the rainy season (B. Mondet et al., unpub. data). Therefore, care should be taken before advising the farmers to avoid small ponds. Beyond the possibly lower risk for RVF, large ponds might be more dangerous for other human and animal diseases such as West Nile fever or schistosomiasis, which are highly prevalent in the Ferlo (*21**–**23*).

Artificial ponds, arranged for their use by livestock, appear to act like large temporary ponds with respect to water-level changes and watering duration. During past years, such ponds were implemented near Barkedji, among other places, and the Senegalese government plans to develop them in the Ferlo. Their impact on human and animal health should be investigated to identify advantages and drawbacks of this possible alternative to the use of natural temporary ponds. Further studies are also needed to assess the influence of ecologic factors on *Aedes* abundance and their relationships to the risk for RVF transmission around the Ferlo temporary ponds.
